# Impact of Promoter Polymorphisms on the Transcriptional Regulation of the Organic Cation Transporter OCT1 (SLC22A1)

**DOI:** 10.3390/jpm8040042

**Published:** 2018-12-11

**Authors:** Kristin Bokelmann, Jürgen Brockmöller, Mladen V. Tzvetkov

**Affiliations:** 1 Institute of Clinical Pharmacology, University Medical Center, Georg-August-University, 37075 Göttingen, Germany; jbrockm@gwdg.de; 2 Institute of Pharmacology, Center of Drug Absorption and Transport (C_DAT), University Medicine Greifswald, 17487 Greifswald, Germany; mladen.tzvetkov@uni-greifswald.de

**Keywords:** OCT1, SNP, promoter, EMSA, luciferase reporter gene assay, expression, NF-Y

## Abstract

The organic cation transporter 1 (OCT1, SLC22A1) is strongly expressed in the human liver and facilitates the hepatic uptake of drugs such as morphine, metformin, tropisetron, sumatriptan and fenoterol and of endogenous substances such as thiamine. OCT1 expression is inter-individually highly variable. Here, we analyzed SNPs in the *OCT1* promoter concerning their potential contribution to the variability in OCT1 expression. Using electrophoretic mobility shift and luciferase reporter gene assays in HepG2, Hep3B, and Huh7 cell lines, we identified the SNPs −1795G>A (rs6935207) and −201C>G (rs58812592) as having effects on transcription factor binding and/or promoter activity. The A-allele of the −1795G>A SNP showed allele-specific binding of the transcription factor NF-Y leading to 2.5-fold increased enhancer activity of the artificial SV40 promoter. However, the −1795G>A SNP showed no significant effects on the native *OCT1* promoter activity. Furthermore, the −1795G>A SNP was not associated with the pharmacokinetics of metformin, fenoterol, sumatriptan and proguanil in healthy individuals or tropisetron efficacy in patients undergoing chemotherapy. Allele-dependent differences in USF1/2 binding and nearly total loss in *OCT1* promoter activity were detected for the G-allele of −201C>G, but the SNP is apparently very rare. In conclusion, common *OCT1* promoter SNPs have only minor effects on OCT1 expression.

## 1. Introduction

The organic cation transporter OCT1 (SLC22A1) is one of the most strongly expressed drug transporters in the human liver [1,2,3]. OCT1 plays a role in the hepatocellular uptake of organic cationic molecules from the blood into the liver. Drugs such as metformin, tramadol, morphine, sumatriptan, tropisetron, fenoterol and proguanil are substrates of OCT1 [4,5,6,7,8,9,10,11].

*OCT1* is genetically highly polymorphic in humans. Resequencing analyses of 2171 unrelated individuals from 67 worldwide populations report 29 variants that cause amino acid substitutions [12]. The variants could be grouped into 30 haplotypes constituting 16 major alleles, which substantially affect OCT1 activity. Due to the presence of these coding polymorphisms and depending on substrate-specific differences of allele OCT1*2, between 3% and 9% of Caucasians have zero or strongly reduced OCT1 activity. This results in decreased hepatic metabolism by affecting their uptake into hepatocytes. These effects have been demonstrated for drugs such as tramadol, debrisoquine, morphine, sumatriptan and tropisetron, and decreased efficacy of metformin in these individuals has been suggested [4,5,6,7,8,13,14]. For numerous other drugs such as lamivudine, bendamustine and debrisoquine, the effects of *OCT1* polymorphisms have been shown *in vitro* or ex *vivo* [13,15,16]. This is now acknowledged as an important cause of variability in the pharmacokinetics and efficacy of drugs [17,18,19]. On the other hand, the tyrosine kinase inhibitors imatinib and sorafenib have been suggested to be affected by *OCT1* polymorphisms, but the data is controversial with some detailed studies that were unable to confirm these drugs as substrates of OCT1 [20,21,22,23].

Beside the coding variations that may strongly affect OCT1 activity, the expression of OCT1 also varies widely among individuals. Nies et al. measured 113-fold variability in *OCT1* mRNA and a corresponding 83-fold variability in OCT1 protein levels [1]. The variability in mRNA expression has been confirmed in further studies [24,25]. Already identified reasons for the variable OCT1 expression, which may cause considerable inter-individual variability of hepatic OCT1 activity, include cholestasis and epigenetic variations [1,26]. 

However, transporter expression, and as a consequence drug pharmacokinetics, may also be affected by single nucleotide polymorphisms (SNPs) in their promoter regions [27,28,29,30,31,32]. Thus, we hypothesize that also *OCT1* promoter polymorphisms, especially in or next to *cis*-regulatory elements, may affect the binding of regulatory factors and *OCT1* gene expression.

The expression of OCT1 is controlled by three transcription factors: USF1/2, HNF4α, and HNF1. The upstream stimulatory factors USF1/2 are binding to an E-box at −200 to −195 and the hepatocyte nuclear factor HNF4α is binding to the two DR2 elements at −1642 to −1604 bp from the transcriptional start site (TSS) [33,34]. Also, the hepatocyte nuclear factor HNF1 has been demonstrated to regulate OCT1 expression by binding to an evolutionary conserved enhancer element in the intron 1 of the *OCT1* gene [24]. 

In the present study, we characterized the functional effects of polymorphisms in the 5 kb upstream region of the *OCT1* gene in order to identify polymorphisms that may contribute to the high variability of OCT1 expression. Therefore, we used electrophoretic mobility shift assays (EMSA) and luciferase reporter gene assays to evaluate the effects of the polymorphisms on promoter activity. We further analyzed those SNPs that showed effects on promoter activity in the *in vitro* assays for associations with pharmacokinetics of metfomin, fenoterol, sumatriptan and proguanil in healthy volunteers and tropisetron efficacy in patients. To the best of our knowledge, these are the first systemic analyses on the effects of single nucleotide polymorphisms on *OCT1* promoter activity.

## 2. Materials and Methods

### 2.1. Cell Culture and Transfection

HepG2 cells (DSMZ-German Collection of Microorganisms and Cell Cultures, Braunschweig, Germany) were cultured in RPMI 1640 GlutaMAX™-I supplemented with 10% fetal bovine serum, 100 U/mL penicillin, and 100 μg/mL streptomycin. Hep-3B cells (DSMZ, Braunschweig, Germany) and Huh7 cells (JCRB Cell Bank, Tokyo, Japan) were cultured in DMEM supplemented with 10% fetal bovine serum, 100 U/mL penicillin, and 100 μg/mL streptomycin. 

Cells were detached with TrypLE Express W/Phenol red (Life Technologies, Darmstadt, Germany). Media and additives were obtained from Gibco (Life Technologies). Cells were cultured under standard conditions at 37 °C in a humidified atmosphere supplemented with 5% CO_2_.

For transfection experiments, 1.5 × 10^5^ Huh7 and Hep-3B cells, respectively, and 2 × 10^5^ HepG2 cells were plated per well of a 12-well plate (Nunc, Langenselbold, Germany) and grown for 24 h to reach approximately 80% confluence. Lipofectamine 2000 (Invitrogen, Karlsruhe, Germany) was used to transfect the cells. Per well, 4 µL Lipofectamine, 1.6 µg plasmid DNA and 3 ng pRL-CMV Renilla luciferase control vector (Promega, Mannheim, Germany) were applied. The cells were harvested and luciferase activity was measured as described previously [24].

### 2.2. Electrophoretic Mobility Shift Assays (EMSA)

Nuclear protein preparation of HepG2, Hep-3B and Huh7 cells was performed according to a protocol for the CelLytic^®^ NuCLEAR^®^ Extraction Kit from Sigma-Aldrich (Deisenhofen, Germany), but with modifications and self-made buffers. Media of 1 × 10^7^–1 × 10^8^ cells was discarded and cells were detached using Tryple Express W/Phenol red. Trypsination was stopped with culture media and cells were transferred into a 50 mL tube and centrifuged at 600× *g* for 3 min at 4 °C. Media was discarded, cells were washed with 10 mL 1× PBS including 1 mM sodium-orthovanadat and centrifuged again. Supernatant was discarded, cells were washed with 1 mL 1× PBS including 1 mM sodium-orthovanadat, transferred into a 2 mL tube and centrifuged again. Then, the volume of the cell pellet was determined and mixed with 5× volume of 1× Lysis buffer (10 mM HEPES, pH 7.9, with 1.5 mM MgCl_2_, 10 mM KCl, 0.5 mM DTT, 1 mM sodium-orthovanadat, 2.5 mM PMSF). Cells were incubated on ice for 30 min and optionally disrupted with a glass tissue homogenizer until the cells were lysed. Then, NP-40 was added to a final concentration of 1% and cells were vortexed vigorously for 10 s. Subsequently, the tubes were centrifuged at 11,000× *g* for 2 min at 4 °C and the supernatant was discarded. The pellet was then resuspended in 2/3 of its volume with the extraction buffer (20 mM HEPES, pH 7.9, with 1.5 mM MgCl_2_, 420 mM NaCl, 0.2 mM EDTA, and 25% (*v*/*v*) Glycerol, 1% NP-40, 0.5 mM DTT, 1 mM sodium-orthovanadat, 2.5 mM PMSF). The tube was mounted on a vortex mixer and agitated at 1800 rpm for 30 min and then centrifuged at 17,000× *g* for 10 min at 4 °C. Optionally, the extraction step could be repeated with 1/3 of the former volume and agitated for 3 h. The supernatant contains the nuclear proteins and was stored at −80 °C. The concentrations of the proteins were assessed by the bicinchoninic acid (Sigma-Aldrich, Deisenhofen, Germany) protein assay with BSA as standard.

Oligonucleotides used for EMSA are shown in Table 1. EMSA was performed as described previously [24]. For supershift assays, a 4% polyacrylamide gel was used and was run for 2 h. Competition assays were performed with specific non-labeled double-stranded oligonucleotide probes in 2- to 15-fold excesses of the labeled probe. For supershift assays, nuclear extracts were incubated with 2 µg antibody (NF-Ya (sc-10779) and IgG (sc-2027), all from Santa Cruz Biotechnology, Heidelberg, Germany) for 1 h on ice, prior to the addition of radiolabeled probes.

### 2.3. Generation of the Luciferase Reporter Plasmids

*OCT1* promoter SNPs were cloned into the pGL3-promoter vector. Therefore, 20 pmol of the appropriate *OCT1* promoter SNP oligonucleotides (Table 1: “EMSA probes”) was annealed. The resulting dsDNA probe carries a *Kpn*I-overhang at the 5′-end and a *Bgl*II-overhang at the 3′-end. The pGL3-promoter vector was cut with *Kpn*I and *Bgl*II, respectively, and ligated with the different dsDNA *OCT1* promoter probes. 

Generation of the pGL3b::OCT1promoter is described in [24]. To also analyze the effect of −1795G>A on the promoter activity, the *OCT1* promoter was extended to −1853 from the translational start site of *OCT1*. Therefore, the adjacent downstream part of the promoter was amplified from gDNA of lymphoblastoid cell lines of homozygote −1795G and −1795A carriers, respectively, with KOD-Polymerase, Q-Solution (Qiagen, Hilden, Germany) and the OCT1-1853bp_promoter primers (Table 1) under the following reaction conditions: 94 °C for 5 min, followed by 35 cycles of 94 °C for 15 s, 57 °C for 30 s, 72 °C for 40 s, and a final elongation of 72 °C for 10 min. The PCR product and pGL3b::OCT1 promoter were cut with *Kpn*I and *Sac*I and afterwards ligated to each other.

Site-directed mutagenesis was used to mutate the wild-types of the SNPs −201C>G and −1620T>C to its variant. The amplification was carried out using the 588mut and 840mut forward and reverse primers, respectively (Table 1) under the following reaction conditions: 95 °C for 3 min, followed by 19 cycles of 95 °C for 30 s, 55 °C for 30 s, and 72 °C for 4 min. Then, 2 µL of the restriction enzyme *Dpn*I was added to the product and incubated for 2 h at 37 °C. Afterwards, it was cloned into *E. coli* TOP10. 

The correct sequences of all plasmids were monitored by restriction analyses and sequencing of the inserts and the flanking regions.

## 3. Results

### 3.1. Screening of SNPs in the OCT1 Promoter for Effects on Transcription Factor Binding and Promoter Activity 

**Figure 1:**
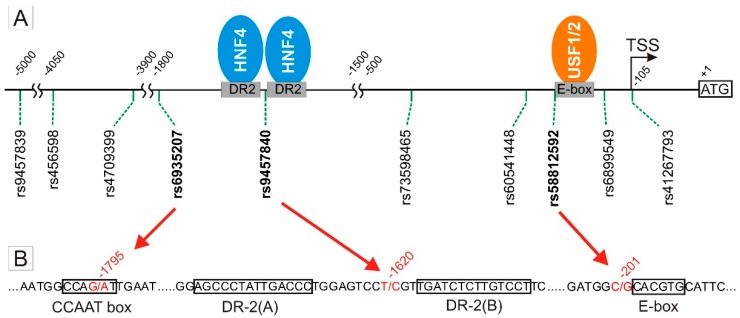
Position of the analyzed *OCT1* polymorphisms. (**A**) The coordinates are given in base pairs relative to the translational start codon ATG (A = +1) of the *OCT1* gene. The transcriptional start site (TSS) is located at −105. The *OCT1* promoter contains two transcriptionally functional *cis*-regulatory elements shown here as gray boxes: a doubled DR2-element at −1642 to −1604 and an E-box at −200 to −195. Binding of the transcription factors HNF4α (blue oval) and USF1/2 (orange oval) to its *cis*-elements regulate OCT1 expression. (**B**) Three SNPs are in special focus: −1795 G>A (rs6935207) shows functional effects and is located within a potential CCAAT-box; −1620 C>G (rs9457840) and −201 T>C (rs58812592) are located in close proximity to the DR2-elements and the E-box, respectively. SNPs and their positions are shown in red; *cis*-regulatory elements are framed.

We analyzed all ten SNPs in the 5 kb upstream region of *OCT1*, which were listed in the NCBI dbSNP database at the time point of the start of this study (Figure 1). 

**Figure 2:**
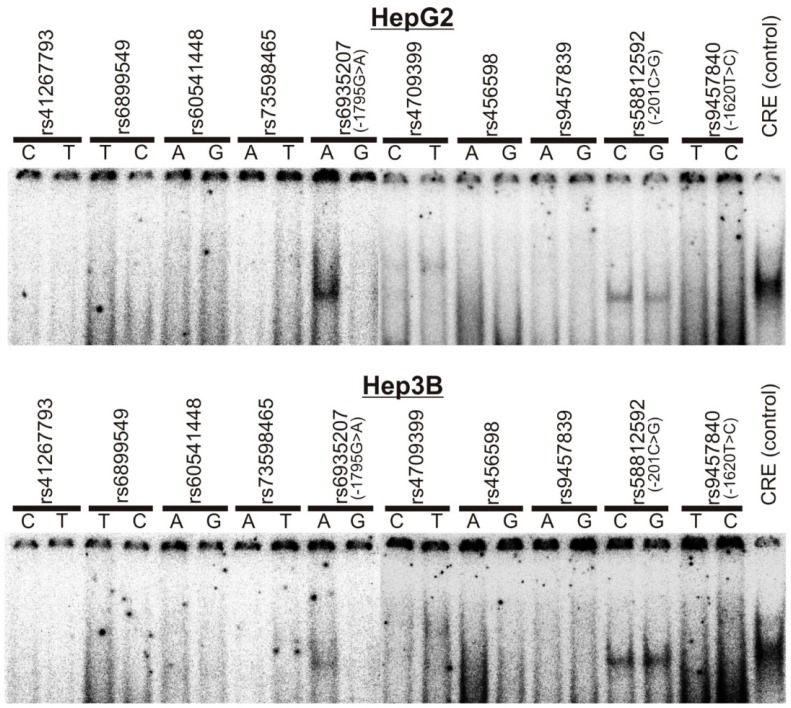
Electrophoretic mobility shift assays with probes carrying *OCT1* promoter SNPs indicate nuclear protein binding for the A-allele of the rs6935207 locus and for both alleles of the rs58812592 locus. The ^32^P-labeled probes, containing ~30 bp around each SNP locus (Table 1), were incubated with nuclear extracts from HepG2 and Hep3B cells and separated on 5% polyacrylamide gels. CRE (=cAMP response element) was used as a positive control for the assay.

First, we performed EMSAs with nuclear proteins of HepG2 and Hep3B cells to screen for transcription factor binding in SNPs’ vicinity (Figure 2). We observed nuclear protein binding for the SNPs −201C>G (rs58812592) and −1795G>A (rs6935207). Thereof, the −1795G>A signal was highly allele-specific. A clear and strong retention signal was detected for the −1795A, but not for the −1795G-allele. 

**Figure 3:**
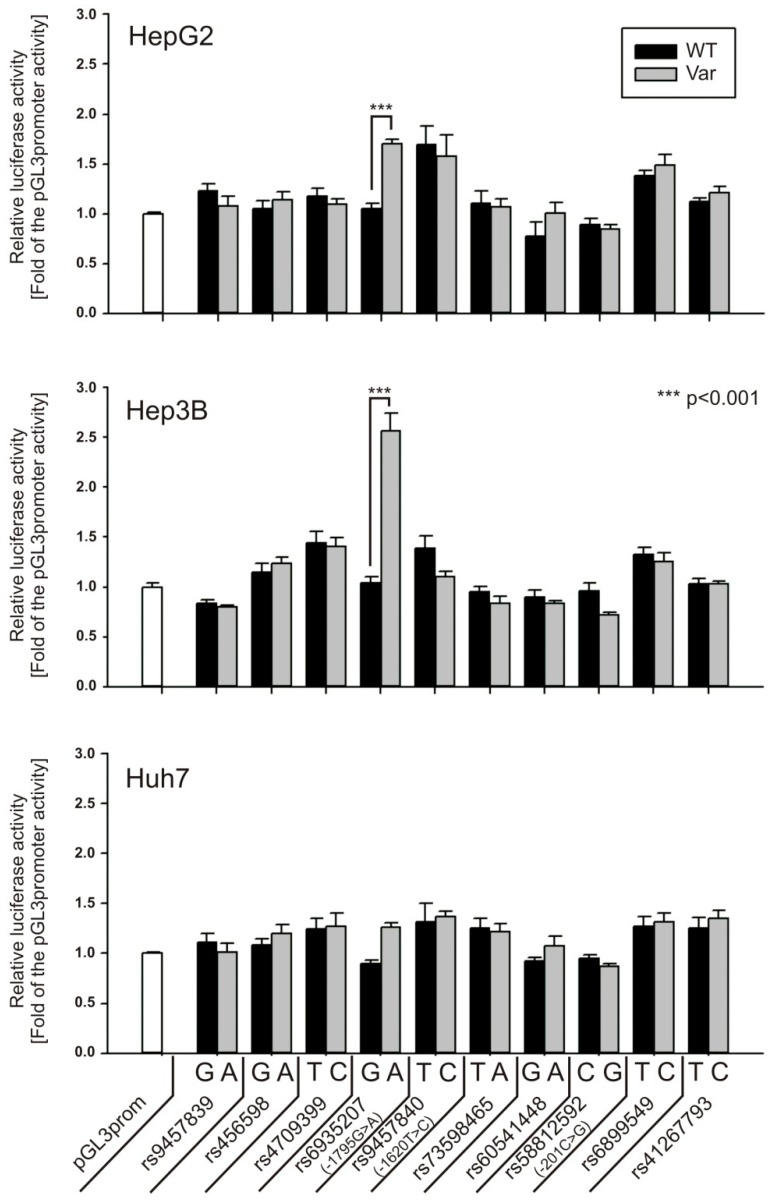
Analysis of the enhancer activity of the *OCT1* promoter SNPs (WT and variant) on a constitutive Simian virus 40 (SV40) promoter. Annealed oligonucleotides harboring ~30 bp around each SNP locus (Table 1) were cloned in front of a constitutive SV40 promoter in the pGL3-promoter vector. Luciferase reporter gene assays were performed in the hepatocellular carcinoma cell lines HepG2, Hep3B and Huh7. The data represent the means and standard deviations of at least two independent experiments conducted in duplicate.

Second, we screened all ten SNPs for their effect on the constitutive SV40 promoter activity (Figure 3). Therefore, we used the same dsDNA probes as before for EMSA, cloned them into the pGL3-promoter vector and performed luciferase reporter gene assays in HepG2, Hep3B and Huh7 cells. Allele-specific differences in SV40 promoter activity were measured only for −1795G>A. The −1795A-allele showed a 2.5-fold (*p* < 0.001) increase in promoter activity in Hep3B, 1.6-fold (*p* < 0.001) increase in HepG2 cells and 1.4-fold (not significant) increase in Huh7 cells, respectively, compared with −1795G. Therefore, we focused our further analysis on −1795 G>A, as well as on −201 C>G (rs58812592) and −1620 T>C (rs9457840) due to their proximity to *cis*-regulatory elements in the *OCT1* promoter.

### 3.2. Specific Binding of NF-Y to the A-Allele of the -1795G>A (rs6935207) Polymorphism

**Figure 4:**
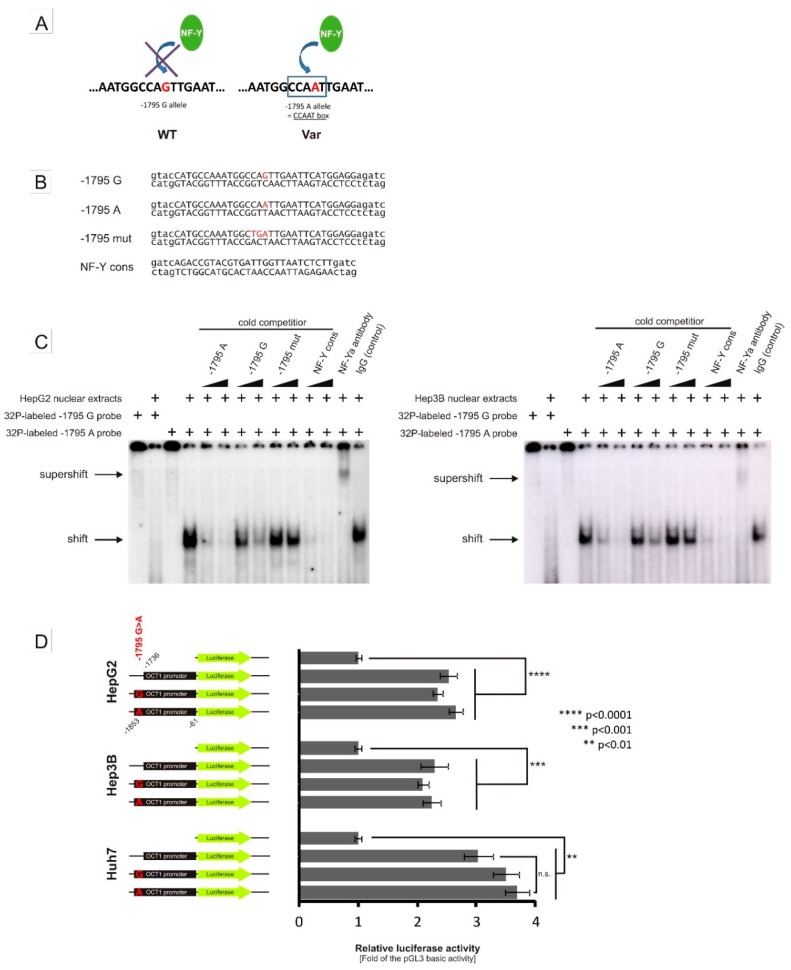
*In vitro* effects of the −1795G>A SNP on the binding of transcription factor NF-Y and *OCT1* promoter activity. (**A**) Model for the allele-specific binding of the transcription factor NF-Y to a CCAAT-box built by the variant A- but not by the wild-type G-allele of −1795 G>A. (**B**) Sequences of the annealed oligonucleotides used as EMSA probes. The specific sequences are given in upper case, and the unspecific sequences used in the radioactive labeling of the EMSA probes are given in lower case letters. The SNP and mutated bases are shown in red. (**C**) A ^32^P-labeled probe containing either a −1795G or −1795A probe was incubated with nuclear extracts from HepG2 (left) and Hep3B cells (right), respectively, in the absence or presence of unlabeled probes (cold competition) or antibodies (supershift). The unlabeled probes were given in 5- and 15-fold molar excess of the ^32^P-labeled probe. (**D**) Luciferase reporter gene assay with the native −1736 and −1853 *OCT1* promoter cloned in front of the luciferase gene in the pGL3-basic vector. The −1853 constructs include either the −1795G or the −1795 A-allele. The coordinates are given in base pairs related to the distance to the translational start site of *OCT1*. The data represent means and standard deviations of at least three independent experiments conducted in duplicate.

Within the probe for the −1795G>A SNP, we detected a potential CCAAT box sequence that was located at −1798bp to −1794 bp and so the −1795A-allele was essential for the CCAAT box consensus (Figure 4A). Cold competition and supershift EMSAs demonstrate the binding of the nuclear transcription factor NF-Y to the sequence containing the −1795A-allele (Figure 4B,C). 

**Figure 5:**
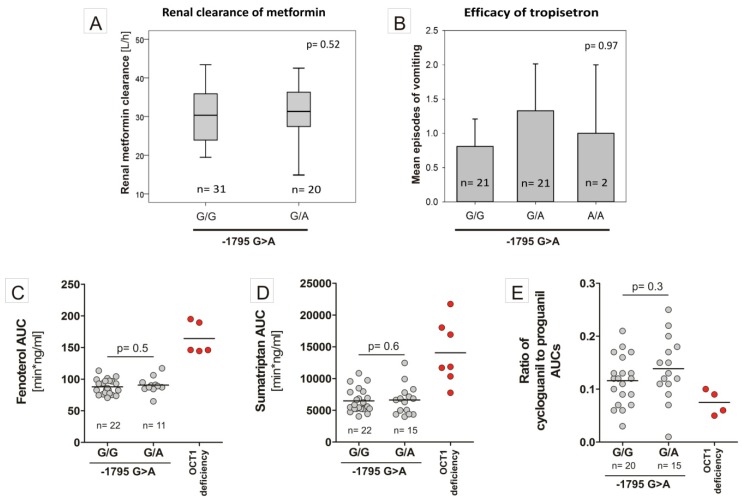
Clinical effects of the −1795G>A SNP. Genotyping was performed using a custom TaqMan^®^ SNP genotyping assay (Thermo Fisher Scientific). (**A**) Renal clearance of metformin in 51 healthy volunteers. (**B**) Efficacy of tropisetron in 45 cancer patients suffering from vomiting. (**C**–**E**) Shown are the area under the time-concentration curves of fenoterol, sumatriptan and the ratio of cycloguanil to proguanil in dependence of the −1795G>A genotype of the volunteers. As comparison, the effects of amino acid mutations resulting in complete loss of OCT1 activity are shown on the right of each chart as red dots. *p*-values are based on non-parametric comparison using the Mann–Whitney-U Test.

With strong evidence for NF-Y binding, leading to an allele-specific increase of activity in the pGL3-promoter constructs, we investigated the effect of −1795G>A on the native *OCT1* promoter. To this end, we cloned the *OCT1* promoter region spanning from −1853 to −61 bp from the translational start codon in the pGL3-basic vector (Figure 4D). In luciferase reporter gene assays, we measured a significant increase in the luciferase activity in wild-type *OCT1* promoter compared to the empty vector in all cell lines tested: 2.34-fold in HepG2 (*p* < 0.0001), 2.10-fold in Hep3B (*p* < 0.001) and 3.51-fold in Huh7 cells (*p* < 0.0001). Surprisingly, there was no difference in the activity for the *OCT1* promoter between the two −1795G>A-alleles. Also, completely omitting the potential NF-Y binding region by using a construct spanning only from −1736 to −61 bp of the *OCT1* promoter did not lead to a significant reduction of the observed promoter activity. Moreover, in clinical human samples (the clinical studies have been described in detail previously [5,6,8,9,11]) we found no effects of −1795G>A on the pharmacokinetics of metformin, fenoterol, sumatriptan and proguanil in healthy volunteers and on the efficacy of tropisetron in cancer patients (Figure 5).

Therefore, despite rather clear results with the artificial SV40 promoter, there is no conclusive evidence that the −1795G>A SNP is relevant for the native *OCT1* promoter activity.

### 3.3. Allele-Dependent Binding of USF1/2 to the −201C>G (rs58812592) Polymorphism

**Figure 6:**
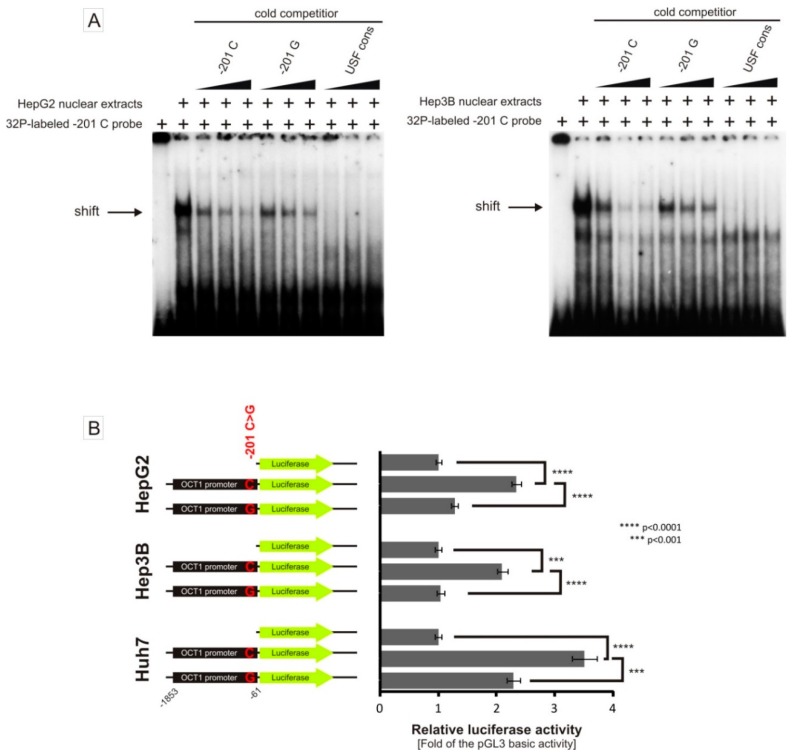
Effects of the −201C>G SNP on binding of the transcription factors USF1/2 and on *OCT1* promoter activity. (**A**) A ^32^P-labeled probe containing the −201C probe was incubated with nuclear extracts from HepG2 and Hep3B cells, respectively, in the absence or presence of unlabeled probes (cold competition) or antibodies (supershift). The unlabeled probes were given in 2-, 4- and 6-fold molar excess of the ^32^P-labeled probe. (**B**) Luciferase reporter gene assay with the native -1853 *OCT1* promoter cloned in front of the luciferase gene in the pGL3-basic vector. The constructs include either the −201C- or the −201 G-allele. The coordinates are given in base pairs related to the distance to the translational start site of *OCT1*. The data represent means and standard deviations of at least three independent experiments conducted in duplicate.

As previously demonstrated by Kajiwara et al. [33], we could confirm binding of USF1/2 to an E-box in the *OCT1* promoter at −200 to −195 from the translational start codon of *OCT1* (Figure 2 and Figure 6A). Furthermore, we analyzed the effect of the −201C>G SNP that is located in close proximity to the E-box (Figure 1). Although, apparently, the intensity of USF1/2 binding to both alleles is quite similar (Figure 2), more detailed cold competition analyses suggest that USF1/2 is binding more strongly to the −201C compared to the −201G-allele (Figure 6A). Moreover, we detected significant allele-specific changes in the native *OCT1* promoter activity in dependence of the −201C>G allele (Figure 6B). However, the −201C>G SNP was monomorphic in our genotyped volunteers and patients and could not be analyzed for clinical effects.

### 3.4. Lack of Effect of -1620T>C on HNF4α Binding and OCT1 Promoter Activity

**Figure 7:**
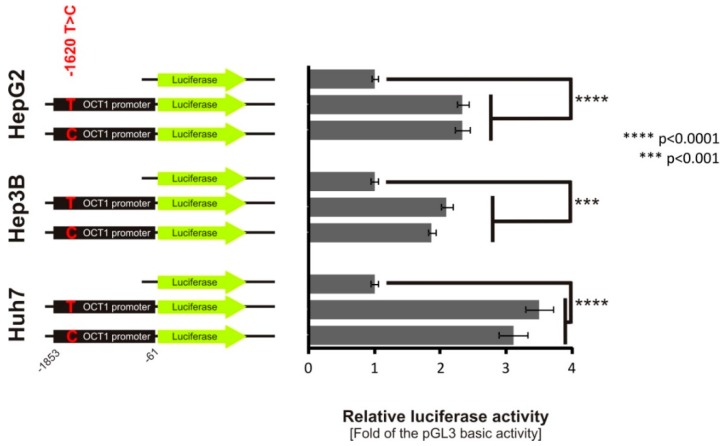
Effect of the −1620 T>C SNP on *OCT1* promoter activity. Luciferase reporter gene assay with the native −1853 *OCT1* promoter cloned in front of the luciferase gene in the pGL3-basic vector. The constructs include either the −1620 T- or the −1620 C-allele. The coordinates are given in base pairs related to the distance to the translational start site of *OCT1*. The data represent means and standard deviations of at least three independent experiments conducted in duplicate.

Despite not detecting the effects of −1620T>C in the functional screens (Figure 2 and Figure 3), we further analyzed this SNP because of its localization between the two transcriptionally important DR2 *cis*-elements in the *OCT1* promoter. However, in line with the lack of retention signal in the EMSA analyses, −1620T>C does not affect native *OCT1* promoter activity in all cell lines tested (Figure 7). 

## 4. Discussion

Here, we analyzed ten polymorphisms in the proximal promoter and up to 5 kb upstream of the human *SLC22A1* gene encoding for the organic cation transporter 1 (OCT1) as a possible source of the known high inter-individual variability in OCT1 expression. We performed functional analyses using luciferase reporter gene and electrophoretic mobility shift assays. The SNPs showing some effect on nuclear protein binding and/or luciferase gene activity were further analyzed for association with the pharmacokinetics parameter, which is known to be limited by OCT1 expression and activity. In contrast to other genes encoding for the important cation transporters OCT2, OCTN2, MATE1 and MATE2-K, where promoter polymorphisms were shown to affect expression and function [29,31,32,35], we were not able to observe the significant effects of common polymorphisms on the expression or activity of OCT1. 

**Figure 8:**
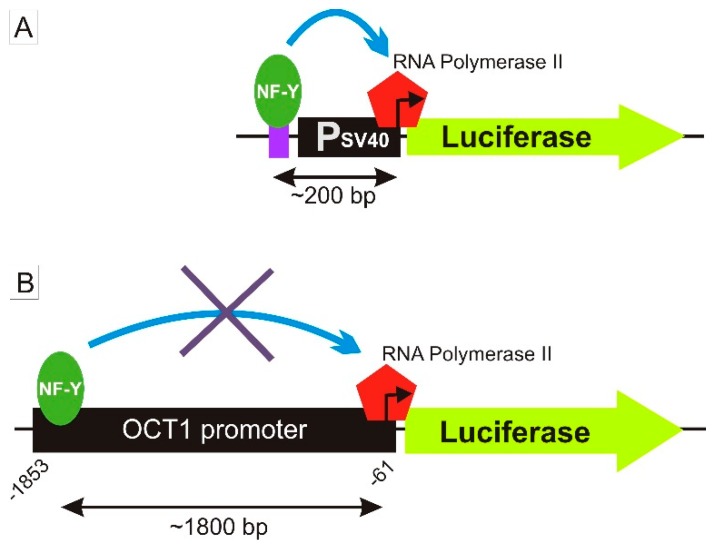
Model for the potential role of the transcription factor NF-Y in an artificial SV40 promoter construct and in the native *OCT1* promoter. (**A**) A possible explanation for the enhancing effect on the SV40 promoter (Figure 3) is that NF-Y binding in close proximity to the TSS contributes to RNA polymerase II recruitment. (**B**) The lack of effect in the native *OCT1* promoter (Figure 4D) may be caused by the large distance between NF-Y and the TSS.

In this study, we found that the nuclear transcription factor Y (NF-Y) may bind to a region −1798 bp to −1794 bp upstream of the start codon of *OCT1* and that this binding may be strongly affected by the common polymorphism −1795G>A (rs6935207). The consensus sequence for NF-Y binding is RRCCAATCA including the essential CCAAT box, which corresponds to the A-allele of the −1795G>A SNP. Indeed, both EMSA and luciferase reporter gene assays show strong functional binding only to the A-allele of this variant (Figure 2, Figure 3 and Figure 4). However, in our study, we detected allele-specific enhancer activity for the region around −1795G>A, but only in the artificially shortened SV40 promoter construct and not in the extended native *OCT1* promoter (Figure 3 and Figure 4). Both, our data (Figure 2, Figure 3 and Figure 4C) and other studies [36,37,38,39,40], showed clear functional activity on the transcription factor NF-Y in HepG2 and Hep3B cells, which we used as models in this study. Therefore, the lack of NF-Y activity in our model could not be an explanation for this observation. One possible explanation is that because of the proximity between the NF-Y binding site and the transcriptional start site (TSS), NF-Y contributes to polymerase II recruitment in the artificial pGL3-promoter construct (Figure 8A). In the native *OCT1* promoter, however, the distance is too large to affect transcription (Figure 8B). Furthermore, the common −1795G>A polymorphism (MAF = 19.7% in Caucasians) neither shows association with renal clearance of metformin or the pharmacokinetics of fenoterol, sumatriptan and proguanil, nor with the efficacy of tropisetron (Figure 5)—parameters known to be dependent on OCT1 activity in humans [5,6,8,9,11,41]. The lack of effects of −1795G>A on OCT1 expression suggested in our work is in line with the observation of Kim et al., who genotyped 65 Koreans and correlated it with genetic and non-genetic factors, but they found no significant associations with any factor (including −1795G>A) to clarify the variability in OCT1 expression [42]. In contrast, Maffioli et al. found a correlation between the −1795G>A SNP with inadequate response to imatinib [43]. However, whether OCT1 plays any role in imatinib response or not is still widely questioned [20,21].

OCT1 expression is known to be regulated by two transcription factors, HNF4α and USF1/2, binding to the *OCT1* promoter, and the transcription factor HNF1 binding to an evolutionary conserved region (ECR) in intron 1 of the *OCT1* gene [24,33,34]. Our analysis of the polymorphism −1620T>C (rs9457840) that is located between the two DR2 elements to which HNF4α is binding, showed no effects on HNF4α binding to the DR2 elements and no effects on SV40 and *OCT1* promoter activity (Figure 3 and Figure 7). However, in the case of a strong overexpression, this effect may change, but is less representative of the *in vivo* conditions in the human liver. As HNF4α expression levels are quite similar in HepG2 and Hep3B cells compared to human liver [44], we kept native HNF4α expression conditions to simulate the native liver environment. Indeed, the polymorphism −201C>G (rs58812592), which is located directly adjacent to the E-box known to be involved in the binding of the transcription factors USF1/2, showed substantial effects on USF1/2 binding and *OCT1* promoter activity (Figure 6), comparable to the effects of a targeted mutation of the E-Box [33]. As binding of USF1/2 to the E-Box is an essential mechanism in the regulation of basal hepatic OCT1 expression, the −201C>G SNP suppresses the transcription of *OCT1*, which would result most likely in decreased OCT1 expression followed by a decreased uptake of drugs into the liver. However, −201C>G is actually a very rare genetic variant. Its minor allele frequency is 0.0001 (TOPMED) and 0.0002 (GnomAD), respectively, according to the dbSNP-database (https://www.ncbi.nlm.nih.gov/snp/rs58812592 accessed on 10th December 2018). This SNP illustrates nicely the potential role of genetic variants affecting the binding of essential transcriptional factors, but cannot explain the commonly observed variation in OCT1 expression. Alternatively, variation in epigenetic [26] or indirect regulation via regulating the expression of key transcriptional factors should be considered [45,46,47].

This study has some limitations. Since we began planning the present study, more and more SNPs were reported in the analyzed 5 kb upstream region of the *OCT1* gene. However, most of the SNPs are very rare or are only found in diverse populations. In total, there are only 11 common SNPs with an MAF of at least 1% in this region (Table S1). Thereof, seven SNPs (inclusive the six most common SNPs) were included in the present study. Additionally, in the ECR in intron 1, contributing to the transcriptional regulation of *OCT1* via HNF1 binding [24], one SNP (rs145668795) had an MAF of at least 1%. 

In conclusion, two out of ten analyzed polymorphisms in the *OCT1* promoter, −1795G>A (rs6935207) and −201C>G (rs58812592), showed functional effects *in vitro*. However, −1795G>A showed only allele-specific effects in EMSA and in an artificial SV40 promoter construct, but not in the native *OCT1* promoter. A possible explanation is that the SNP affects the binding of the transcriptional factor NF-Y, but due to the large distance to the transcriptional start site, this binding may not affect the native *OCT1* promoter activity. Furthermore, we identified the SNP −201C>G to be causing strong functional effects in the native *OCT1* promoter by affecting the binding of the transcription factor USF1/2. Although extremely rare, if present, this SNP may strongly influence OCT1 expression. Finally, none of the analyzed common SNPs in the promoter region of *OCT1* could explain the high inter-individual variability in OCT1 expression.

## Figures and Tables

**Table 1:** Oligonucleotides used in this study.

Name	Direction	Sequence
EMSA Probes *		
rs41267793_T_f	Forward	5′-ACTTGGACAGCAAACTGATTTCAAACCACTCa-3′
rs41267793_T_r	Reverse	5′-gatctGAGTGGTTTGAAATCAGTTTGCTGTCCAAGTgtac-3′
rs41267793_C_f	Forward	5′-ACTTGGACAGCAAACCGATTTCAAACCACTCa-3′
rs41267793_C_r	Reverse	5′-gatctGAGTGGTTTGAAATCGGTTTGCTGTCCAAGTgtac-3′
rs6899549_T_f	Forward	5′-AGGGTAAAAGATTATTTCTACTTGGTTGCCTa-3′
rs6899549_T_r	Reverse	5′-gatctAGGCAACCAAGTAGAAATAATCTTTTACCCTgtac-3′
rs6899549_C_f	Forward	5′-AGGGTAAAAGATTATCTCTACTTGGTTGCCTa-3′
rs6899549_C_r	Reverse	5′-gatctAGGCAACCAAGTAGAGATAATCTTTTACCCTgtac-3′
rs58812592_C_f	Forward	5′-TTGATCAGATGGCCACGTGCATTCTTCCTTTa-3′
rs58812592_C_r	Reverse	5′-gatctAAAGGAAGAATGCACGTGGCCATCTGATCAAgtac-3′
rs58812592_G_f	Forward	5′-TTGATCAGATGGGCACGTGCATTCTTCCTTTa-3′
rs58812592_G_r	Reverse	5′-gatctAAAGGAAGAATGCACGTGCCCATCTGATCAAgtac-3′
rs60541448_G_f	Forward	5′-CACTGACTCGCTCCCGGGCAAAGCAAACGATa-3′
rs60541448_G_r	Reverse	5′-gatctATCGTTTGCTTTGCCCGGGAGCGAGTCAGTGgtac-3′
rs60541448_A_f	Forward	5′-CACTGACTCGCTCCCAGGCAAAGCAAACGATa-3′
rs60541448_A_r	Reverse	5′-gatctATCGTTTGCTTTGCCTGGGAGCGAGTCAGTGgtac-3′
rs73598465_T_f	Forward	5′-TATCACAGAACTAATTAGCCGAATACAGTATa-3′
rs73598465_T_r	Reverse	5′-gatctATACTGTATTCGGCTAATTAGTTCTGTGATAgtac-3′
rs73598465_A_f	Forward	5′-TATCACAGAACTAATAAGCCGAATACAGTATa-3′
rs73598465_A_r	Reverse	5′-gatctATACTGTATTCGGCTTATTAGTTCTGTGATAgtac-3′
rs9457840_T_f	Forward	5′-GGAGCCCTATTGACCCTGGAGTCCTGTTGATCTCTTGTCCTTCa-3′
rs9457840_T_r	Reverse	5′-gatctGAAGGACAAGAGATCAACAGGACTCCAGGGTCAATAGGGCTCCgtac-3′
rs9457840_C_f	Forward	5′-GGAGCCCTATTGACCCTGGAGTCCCGTTGATCTCTTGTCCTTCa-3′
rs9457840_C_r	Reverse	5′-gatctGAAGGACAAGAGATCAACGGGACTCCAGGGTCAATAGGGCTCCgtac-3′
rs6935207_G_f	Forward	5′-CATGCCAAATGGCCAGTTGAATTCATGGAGGa-3′
rs6935207_G_r	Reverse	5′-gatctCCTCCATGAATTCAACTGGCCATTTGGCATGgtac-3′
rs6935207_A_f	Forward	5′-CATGCCAAATGGCCAATTGAATTCATGGAGGa-3′
rs6935207_A_r	Reverse	5′-gatctCCTCCATGAATTCAATTGGCCATTTGGCATGgtac-3′
rs6935207_mut_f	Forward	5′-CATGCCAAATGGC**TG**ATTGAATTCATGGAGGa-3′
rs6935207_mut_r	Reverse	5′-gatctCCTCCATGAATTCAAT**CA**GCCATTTGGCATGgtac-3′
rs4709399_T_f	Forward	5′-AAACCTCTGTGGTCATGGTGCCTTTGCATGAa-3′
rs4709399_T_r	Reverse	5′-gatctTCATGCAAAGGCACCATGACCACAGAGGTTTgtac-3′
rs4709399_C_f	Forward	5′-AAACCTCTGTGGTCACGGTGCCTTTGCATGAa-3′
rs4709399_C_r	Reverse	5′-gatctTCATGCAAAGGCACCGTGACCACAGAGGTTTgtac-3′
rs456598_G_f	Forward	5′-GTAAGGCACTTTTTGGATGGTAGGACTGGTTa-3′
rs456598_G_r	Reverse	5′-gatctAACCAGTCCTACCATCCAAAAAGTGCCTTACgtac-3′
rs456598_A_f	Forward	5′-GTAAGGCACTTTTTGAATGGTAGGACTGGTTa-3′
rs456598_A_r	Reverse	5′-gatctAACCAGTCCTACCATTCAAAAAGTGCCTTACgtac-3′
rs9457839_G_f	Forward	5′-CCACATGCACTCCTAGGTCTGAAAATGGGGGa-3′
rs9457839_G_r	Reverse	5′-gatctCCCCCATTTTCAGACCTAGGAGTGCATGTGGgtac-3′
rs9457839_A_f	Forward	5′-CCACATGCACTCCTAAGTCTGAAAATGGGGGa-3′
rs9457839_A_r	Reverse	5′-gatctCCCCCATTTTCAGACTTAGGAGTGCATGTGGgtac-3′
GS_NF-Y_f	Forward	5′-GATCAGACCGTACGTGATTGGTTAATCTCTT-3′
GS_NF-Y_r	Reverse	5′-GATCAAGAGATTAACCAATCACGTACGGTCT-3′
CRE2_f	Forward	5′-actggTCCTTGGCTGACGTCAGAGAGAGAG-3′
CRE_r	Reverse	5′-taCTCTCTCTCTGACGTCAGCCAAGGAgg-3′
**Primers used for site-directed mutagenesis**		
588_mut_f	Forward	5′-GATTTGATCAGATGG**G**CACGTGCATTCTTCC-3′
588_mut_r	Reverse	5′-GGAAGAATGCACGTG**C**CCATCTGATCAAATC-3′
840_mut_f	Forward	5′-TTGACCCTGGAGTCC**C**GTTGATCTCTTGTCC-3′
840_mut_r	Reverse	5′-GGACAAGAGATCAAC**G**GGACTCCAGGGTCAA-3′
**Primers used for Sanger sequencing**		
Seq_840_f	Forward	5′-TGCAACCAGTTTGCACAGAGAG-3′
Seq_588_f	Forward	5′-GCCTCATACCATCACATCTAGA-3′
Seq_pGL3prom_f	Forward	5′-GAATCGATAGTACTAACATA-3′
Seq_SV40_r	Reverse	5′-AAGCCTCCTCACTACTTCTG-3′
**Primers used for generation of the reporter gene construct**		
OCT1-1853bp_promoter_f	Forward	5′-ATCGCGGTACCTTTTTAAGAAGTCCTTTTAAGT-3′
OCT1-1853bp_promoter_r	Reverse	5′-AAGAAGGGAAGGACAAGAGATCAAC-3′

In the EMSA probes, the unspecific sequences used in the radioactive labeling are given in lowercase letters; the SNPs are highlighted in gray. Mutated nucleotides are shown in boldface, and the artificially introduced restriction site is underlined. * The sequences used as EMSA probes were also used for the SV40 promoter-based reporter assays.

## Supplementary Materials

The following are available online at http://www.mdpi.com/2075-4426/8/4/42/s1, Table S1: Common SNPs (MAF > 1%) in the 5 kb upstream region and in the ECR in intron 1 of the *OCT1* gene.

## References

[1] Nies A.T., Koepsell H., Winter S., Burk O., Klein K., Kerb R., Zanger U.M., Keppler D., Schwab M., Schaeffeler E. (2009). Expression of organic cation transporters OCT1 (SLC22A1) and OCT3 (SLC22A3) is affected by genetic factors and cholestasis in human liver. Hepatology.

[2] Schaefer O., Ohtsuki S., Kawakami H., Inoue T., Liehner S., Saito A., Sakamoto A., Ishiguro N., Matsumaru T., Terasaki T. (2012). Absolute quantification and differential expression of drug transporters, cytochrome p450 enzymes, and UDP-glucuronosyltransferases in cultured primary human hepatocytes. Drug Metab. Dispos..

[3] Wang L., Prasad B., Salphati L., Chu X., Gupta A., Hop C.E., Evers R., Unadkat J.D. (2015). Interspecies variability in expression of hepatobiliary transporters across human, dog, monkey, and rat as determined by quantitative proteomics. Drug Metab. Dispos..

[4] Tzvetkov M.V., Saadatmand A.R., Lotsch J., Tegeder I., Stingl J.C., Brockmoller J. (2011). Genetically polymorphic oct1: Another piece in the puzzle of the variable pharmacokinetics and pharmacodynamics of the opioidergic drug tramadol. Clin. Pharmacol. Ther..

[5] Tzvetkov M.V., Vormfelde S.V., Balen D., Meineke I., Schmidt T., Sehrt D., Sabolic I., Koepsell H., Brockmoller J. (2009). The effects of genetic polymorphisms in the organic cation transporters OCT1, OCT2, and OCT3 on the renal clearance of metformin. Clin. Pharmacol. Ther..

[6] Tzvetkov M.V., Saadatmand A.R., Bokelmann K., Meineke I., Kaiser R., Brockmoller J. (2012). Effects of OCT1 polymorphisms on the cellular uptake, plasma concentrations and efficacy of the 5-HT(3) antagonists tropisetron and ondansetron. Pharmacogenomics J..

[7] Tzvetkov M.V., dos Santos Pereira J.N., Meineke I., Saadatmand A.R., Stingl J.C., Brockmoller J. (2013). Morphine is a substrate of the organic cation transporter OCT1 and polymorphisms in *OCT1* gene affect morphine pharmacokinetics after codeine administration. Biochem. Pharmacol..

[8] Matthaei J., Kuron D., Faltraco F., Knoch T., Dos Santos Pereira J.N., Abu Abed M., Prukop T., Brockmoller J., Tzvetkov M.V. (2016). OCT1 mediates hepatic uptake of sumatriptan and loss-of-function OCT1 polymorphisms affect sumatriptan pharmacokinetics. Clin. Pharmacol. Ther..

[9] Tzvetkov M.V., Matthaei J., Pojar S., Faltraco F., Vogler S., Prukop T., Seitz T., Brockmoller J. (2018). Increased systemic exposure and stronger cardiovascular and metabolic adverse reactions to fenoterol in individuals with heritable oct1 deficiency. Clin. Pharmacol. Ther..

[10] Sundelin E., Gormsen L.C., Jensen J.B., Vendelbo M.H., Jakobsen S., Munk O.L., Christensen M., Brosen K., Frokiaer J., Jessen N. (2017). Genetic polymorphisms in organic cation transporter 1 attenuates hepatic metformin exposure in humans. Clin. Pharmacol. Ther..

[11] Matthaei J., Seitz T., Jensen O., Tann A., Prukop T., Tadjerpisheh S., Brockmoller J., Tzvetkov M.V. (2018). Oct1 deficiency affects hepatocellular concentrations and pharmacokinetics of cycloguanil, the active metabolite of the antimalarial drug proguanil. Clin. Pharmacol. Ther..

[12] Seitz T., Stalmann R., Dalila N., Chen J., Pojar S., Dos Santos Pereira J.N., Kratzner R., Brockmoller J., Tzvetkov M.V. (2015). Global genetic analyses reveal strong inter-ethnic variability in the loss of activity of the organic cation transporter oct1. Genome Med..

[13] Saadatmand A.R., Tadjerpisheh S., Brockmoller J., Tzvetkov M.V. (2012). The prototypic pharmacogenetic drug debrisoquine is a substrate of the genetically polymorphic organic cation transporter OCT1. Biochem. Pharmacol..

[14] Shu Y., Sheardown S.A., Brown C., Owen R.P., Zhang S., Castro R.A., Ianculescu A.G., Yue L., Lo J.C., Burchard E.G. (2007). Effect of genetic variation in the organic cation transporter 1 (OCT1) on metformin action. J. Clin. Investig..

[15] Arimany-Nardi C., Minuesa G., Keller T., Erkizia I., Koepsell H., Martinez-Picado J., Pastor-Anglada M. (2016). Role of human organic cation transporter 1 (hOCT1) polymorphisms in lamivudine (3TC) uptake and drug-drug interactions. Front. Pharmacol..

[16] Arimany-Nardi C., Montraveta A., Lee-Verges E., Puente X.S., Koepsell H., Campo E., Colomer D., Pastor-Anglada M. (2015). Human organic cation transporter 1 (hOCT1) as a mediator of bendamustine uptake and cytotoxicity in chronic lymphocytic leukemia (CLL) cells. Pharmacogenomics J..

[17] Zamek-Gliszczynski M.J., Giacomini K.M., Zhang L. (2018). Emerging clinical importance of hepatic organic cation transporter 1 (OCT1) in drug pharmacokinetics, dynamics, pharmacogenetic variability, and drug interactions. Clin. Pharmacol. Ther..

[18] Tzvetkov M.V. (2017). OCT1 pharmacogenetics in pain management: Is a clinical application within reach?. Pharmacogenomics.

[19] Yee S.W., Brackman D.J., Ennis E.A., Sugiyama Y., Kamdem L.K., Blanchard R., Galetin A., Zhang L., Giacomini K.M. (2018). Influence of transporter polymorphisms on drug disposition and response: A perspective from the international transporter consortium. Clin. Pharmacol. Ther..

[20] Nies A.T., Schaeffeler E., van der Kuip H., Cascorbi I., Bruhn O., Kneba M., Pott C., Hofmann U., Volk C., Hu S. (2014). Cellular uptake of imatinib into leukemic cells is independent of human organic cation transporter 1 (OCT1). Clin Cancer Res..

[21] Tzvetkov M.V., Seitz T., Bokelmann K., Mueller T., Brockmoller J., Koepsell H. (2014). Does the haplotype Met408-Del420, which was apparently predictive for imatinib efficacy, really exist and how strongly may it affect oct1 activity?. Blood.

[22] Neul C., Baker S.D., Sparreboom A., Schaeffeler E., Laufer S., Schwab M., Nies A.T. Evaluation of organic cation transporter 1 (OCT1, SLC22A1) as transporter for sorafenib. Proceedings of the AACR 107th Annual Meeting 2016.

[23] Neul C., Schaeffeler E., Laufer S., Schwab M., Nies A.T. Cellular uptake of sorafenib is independent of major human organic cation and organic anion uptake transporters of the hepatocyte. Proceedings of the 17th Jahreskongress für Klinische Pharmakologie.

[24] O’Brien V.P., Bokelmann K., Ramirez J., Jobst K., Ratain M.J., Brockmoller J., Tzvetkov M.V. (2013). Hepatocyte nuclear factor 1 regulates the expression of the organic cation transporter 1 via binding to an evolutionary conserved region in intron 1 of the oct1 gene. J. Pharmacol. Exp. Ther..

[25] Fattah S., Shinde A.B., Matic M., Baes M., van Schaik R.H.N., Allegaert K., Parmentier C., Richert L., Augustijns P., Annaert P. (2017). Inter-subject variability in OCT1 activity in 27 batches of cryopreserved human hepatocytes and association with oct1 mrna expression and genotype. Pharm. Res..

[26] Schaeffeler E., Hellerbrand C., Nies A.T., Winter S., Kruck S., Hofmann U., van der Kuip H., Zanger U.M., Koepsell H., Schwab M. (2011). DNA methylation is associated with downregulation of the organic cation transporter OCT1 (SLC22A1) in human hepatocellular carcinoma. Genome Med..

[27] Hesselson S.E., Matsson P., Shima J.E., Fukushima H., Yee S.W., Kobayashi Y., Gow J.M., Ha C., Ma B., Poon A. (2009). Genetic variation in the proximal promoter of ABC and SLC superfamilies: Liver and kidney specific expression and promoter activity predict variation. PLoS ONE.

[28] Chen L., Hong C., Chen E.C., Yee S.W., Xu L., Almof E.U., Wen C., Fujii K., Johns S.J., Stryke D. (2013). Genetic and epigenetic regulation of the organic cation transporter 3, *SLC22A3*. Pharmacogenomics J..

[29] Ogasawara K., Terada T., Motohashi H., Asaka J., Aoki M., Katsura T., Kamba T., Ogawa O., Inui K. (2008). Analysis of regulatory polymorphisms in organic ion transporter genes (SLC22A) in the kidney. J. Hum. Genet..

[30] Chung J.Y., Cho S.K., Kim T.H., Kim K.H., Jang G.H., Kim C.O., Park E.M., Cho J.Y., Jang I.J., Choi J.H. (2013). Functional characterization of MATE2-K genetic variants and their effects on metformin pharmacokinetics. Pharmacogenet. Genom..

[31] Choi J.H., Yee S.W., Ramirez A.H., Morrissey K.M., Jang G.H., Joski P.J., Mefford J.A., Hesselson S.E., Schlessinger A., Jenkins G. (2011). A common 5′-UTR variant in MATE2-K is associated with poor response to metformin. Clin. Pharmacol. Ther..

[32] Ha Choi J., Wah Yee S., Kim M.J., Nguyen L., Ho Lee J., Kang J.O., Hesselson S., Castro R.A., Stryke D., Johns S.J. (2009). Identification and characterization of novel polymorphisms in the basal promoter of the human transporter, mate1. Pharmacogenet. Genom..

[33] Kajiwara M., Terada T., Asaka J., Aoki M., Katsura T., Ikai I., Inui K. (2008). Regulation of basal core promoter activity of human organic cation transporter 1 (OCT1/SLC22A1). Am. J. Physiol. Gastrointest. Liver Physiol..

[34] Saborowski M., Kullak-Ublick G.A., Eloranta J.J. (2006). The human organic cation transporter-1 gene is transactivated by hepatocyte nuclear factor-4aα. J. Pharmacol. Exp. Ther..

[35] Tahara H., Yee S.W., Urban T.J., Hesselson S., Castro R.A., Kawamoto M., Stryke D., Johns S.J., Ferrin T.E., Kwok P.Y. (2009). Functional genetic variation in the basal promoter of the organic cation/carnitine transporters OCTN1 (SLC22A4) and OCTN2 (SLC22A5). J. Pharmacol. Exp. Ther..

[36] Zhang Y., Chen B., Li Y., Chen J., Lou G., Chen M., Zhou D. (2008). Transcriptional regulation of the human PNRC promoter by nfy in HEPG2 cells. J. Biochem..

[37] Novak E.M., Bydlowski S.P. (1997). NFY transcription factor binds to regulatory element AIC and transactivates the human apolipoprotein A-I promoter in HEPG2 cells. Biochem. Biophys. Res. Commun..

[38] Pallai R., Simpkins H., Chen J., Parekh H.K. (2010). The CCAAT box binding transcription factor, nuclear factor-Y (NF-Y) regulates transcription of human aldo-keto reductase 1C1 (AKR1C1) gene. Gene.

[39] Yanagawa Y., Chen J.C., Hsu L.C., Yoshida A. (1995). The transcriptional regulation of human aldehyde dehydrogenase I gene. The structural and functional analysis of the promoter. J. Biol. Chem..

[40] Ueda A., Yoshimura T. (1996). Characterization of *cis*-acting elements of the gene for *macrophage-stimulating protein* from the human. The involvement of positive and negative regulatory elements. J. Biol. Chem..

[41] Christensen M.M., Brasch-Andersen C., Green H., Nielsen F., Damkier P., Beck-Nielsen H., Brosen K. (2011). The pharmacogenetics of metformin and its impact on plasma metformin steady-state levels and glycosylated hemoglobin A1c. Pharmacogenet. Genom..

[42] Kim M.H., Shin H.J., Lim S.J., Park J.S., Lee S.S., Song I.S., Shin J.G. (2012). Inter-individual variability in OCT1 expression and its relationship with OCT1 genotype in liver samples from a korean population. Drug Metab. Pharmacokinet..

[43] Maffioli M., Camos M., Gaya A., Hernandez-Boluda J.C., Alvarez-Larran A., Domingo A., Granell M., Guillem V., Vallansot R., Costa D. (2011). Correlation between genetic polymorphisms of the hOCT1 and mdr1 genes and the response to imatinib in patients newly diagnosed with chronic-phase chronic myeloid leukemia. Leuk. Res..

[44] Martinez-Jimenez C.P., Gomez-Lechon M.J., Castell J.V., Jover R. (2006). Underexpressed coactivators PGC1α and SRC1 impair hepatocyte nuclear factor 4α function and promote dedifferentiation in human hepatoma cells. J. Biol. Chem..

[45] Hyrsova L., Smutny T., Carazo A., Moravcik S., Mandikova J., Trejtnar F., Gerbal-Chaloin S., Pavek P. (2016). The pregnane x receptor down-regulates organic cation transporter 1 (SLC22A1) in human hepatocytes by competing for (“squelching”) SRC-1 coactivator. Br. J. Pharmacol..

[46] Rulcova A., Krausova L., Smutny T., Vrzal R., Dvorak Z., Jover R., Pavek P. (2013). Glucocorticoid receptor regulates organic cation transporter 1 (OCT1, SLC22A1) expression via hnf4alpha upregulation in primary human hepatocytes. Pharmacol. Rep..

[47] Hyrsova L., Smutny T., Trejtnar F., Pavek P. (2016). Expression of organic cation transporter 1 (OCT1): Unique patterns of indirect regulation by nuclear receptors and hepatospecific gene regulation. Drug Metab. Rev..

