# Supplementary material for: Impact of Promoter Polymorphisms on the Transcriptional Regulation of the Organic Cation Transporter OCT1 (SLC22A1)
Source: J Pers Med. 2018 Dec 11;8(4):42. doi: 10.3390/jpm8040042 (PMC6313513; doi:10.3390/jpm8040042)
Supplement: Supplementary file 1 [file jpm-08-00042-s001.pdf]

Supplementary

Table S1. Common SNPs (MAF > 1%) in the 5 kb upstream region and in the ECR in intron 1 of the *OCT1* gene.

SNP ID	MAF	Position to ATG	WT	Var
rs4709399	0.43211	-3922	C	T
rs6935207	0.35304	-1795	G	A
rs456598	0.09844	-4047	G	A
rs9457840	0.07768	-1620	T	C
rs9457839	0.07089	-4948	G	A
rs73598465	0.04992	-430	T	A
rs79951329	0.03215	-3941	C	T
rs75332505	0.03215	-3638	T	A
rs12110656	0.03195	-1419	T	A
rs6899549	0.01917	-149	T	C
rs57504133	0.01857	-1198	A	G
rs145668795	0.01458	+3300	T	A

SNPs are sorted by the highest MAF. SNPs that are analyzed in this study are shown in green; SNPs that are not analyzed are shown in yellow (within 5 kb upstream region) and orange (within the ECR in intron 1).
